# Heat Shock Proteins in Cancer Immunotherapy

**DOI:** 10.1155/2019/3267207

**Published:** 2019-12-11

**Authors:** Jugal Kishore Das, Xiaofang Xiong, Xingcong Ren, Jin-Ming Yang, Jianxun Song

**Affiliations:** ^1^Department of Microbial Pathogenesis and Immunology, Texas A&M University Health Science Center, Bryan, TX 77807, USA; ^2^Department of Toxicology and Cancer Biology, University of Kentucky College of Medicine, Lexington, KY 40536, USA

## Abstract

Heat shock proteins (HSPs) are highly conserved molecular chaperones with divergent roles in various cellular processes. The HSPs are classified according to their molecular size as HSP27, HSP40, HSP60, HSP70, and HSP90. The HSPs prevent nonspecific cellular aggregation of proteins by maintaining their native folding energetics. The disruption of this vital cellular process, driven by the aberrant expression of HSPs, is implicated in the progression of several different carcinomas. Many HSPs are also actively involved in promoting the proliferation and differentiation of tumor cells, contributing to their metastatic phenotype. Upregulation of these HSPs is associated with the poor outcome of anticancer therapy in clinical settings. On the other hand, these highly expressed HSPs may be exploited as viable immunotherapeutic targets for different types of cancers. This review discusses recent advances and perspectives on the research of HSP-based cancer immunotherapy.

## 1. Introduction

Cells respond to stressful conditions by activating stress response proteins that promote cellular sustenance. Heat shock proteins (HSPs) are highly conserved stress response chaperone proteins, which are synthesized in response to various stresses. These HSPs have cryoprotective and other critical cytoprotective functions. The ability of the HSPs to protect cells from damaging stress has been attributed to their chaperoning activity through which they prevent misfolding and expedite the refolding and renaturation of proteins [1, 2]. However, when reaching the limit of stress tolerance, the cells invoke programmed cell death (apoptosis or autophagy) to prevent irrevocable systemic damage to the organism. HSPs also play critical roles in inhibiting proapoptogenic molecules through modulation of several signaling cascades such as JNK, AKT, and NF-*κ*B [3]. The HSPs are therefore at the core of maintaining a fine balance between cell death and survival, significantly impacting the biological consequences. The overwhelming evidence on the emerging role of HSPs in modulating carcinogenesis has precisely extended their relevance from simple diagnostic biomarkers to central targets in cancer therapeutics.

## 2. Role of HSPs in Cancer

HSPs are highly expressed in various types of carcinomas. The levels of circulating HSPs along with the antibodies to HSPs are excellent biomarkers for analyzing the stage and aggressiveness of certain types of cancer. HSPs are implicated in tumor cell proliferation, differentiation, invasion, and metastasis [4]. Some HSPs like HSP27 have been shown to contribute to the poor prognosis of osteosarcomas and gastric carcinomas [5]. HSP70 has been found to significantly influence the prognosis of breast cancer [6]. Expressions of HSP27 and HSP70 were reported to affect the response of tumor cells to conventional anticancer treatment [7, 8]. For instance, increased expression of HSP27 leads to poor outcome of chemotherapy in breast cancer and leukemia patients; by contrast, increased expression of HSP70 in osteosarcomas results in an improvement of the outcome of chemotherapy [9]. HSP60 is another key heat shock chaperonin protein which predominantly localizes in the mitochondria and aids in the folding and transport of mitochondrial proteins. HSP60 has also been implicated in the progression of cancer, as the levels and the cellular localization of HSP60 were found to be altered in several different carcinomas [10]. The HSP60 chaperonopathic carcinomas could be inherited or acquired, in which this chaperone plays a significant etiologic-pathogenic role. Like the other HSPs, the high-molecular weight HSP90 chaperone is also a significant regulator of the process of tumor progression. The association of HSP90 with its client proteins has been explored extensively in cancer research. Several inhibitors of HSP90 have shown to be very effective against carcinogenesis [11, 12].

**Figure 1:**
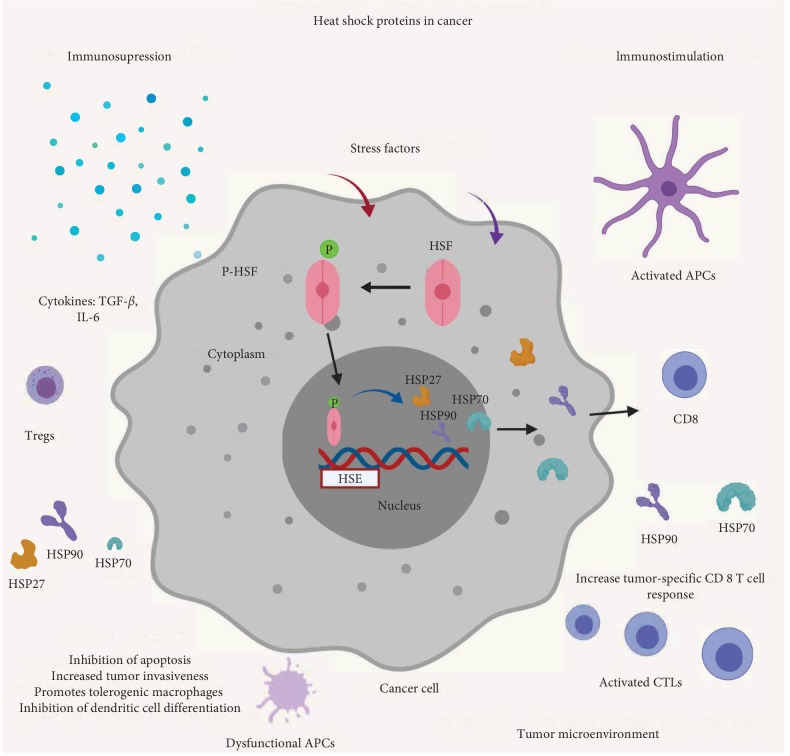
Heat shock proteins in cancer. Cancer cells are exposed to several stress factors from the extracellular milieu of tumor microenvironment. These stress factors activate heat shock transcription factors (HSFs) by facilitating their dissociation from heat shock proteins and phosphorylating them. The heat shock transcription factors are then translocated into the nucleus where they bind with heat shock elements (HSE) and initiate the transcription of heat shock proteins like HSP27, HSP70, and HSP90. The HSPs are exported into the tumor microenvironment modulating the immune response against cancer cells. In immunosuppressive conditions, the HSPs enhance the survival and proliferation of cancer cells by activating their cellular protection machinery. The HSPs may also stimulate the anticancer immune response under optimal conditions, thereby maintaining a fine balance between cell death and survival.

HSPs are not only involved in tumor progression but also in determining their response to treatment. Some of the critical HSPs intricately regulate the fine balance between the protective and destructive immunological responses within the tumor microenvironment (Figure 1), thereby making it imperative to comprehend their central roles in oncoimmunology. Modulating the expression or activity of HSP chaperones has been explored as an anticancer therapeutic strategy. The use of HSP-based immunotherapeutic approaches and development of anticancer vaccines have also greatly contributed to the enrichment of the field of oncoimmunology and assisted in devising more effective anticancer regimens. Cancer immunotherapy adopts a number of approaches including, but not limited to, targeted antibody therapies, immune-checkpoint inhibitors, cytokines, and adoptive cell transfer methodologies. HSPs are now considered as viable targets for cancer immunotherapy.

## 3. HSPs as Immunomodulants

**Figure 2:**
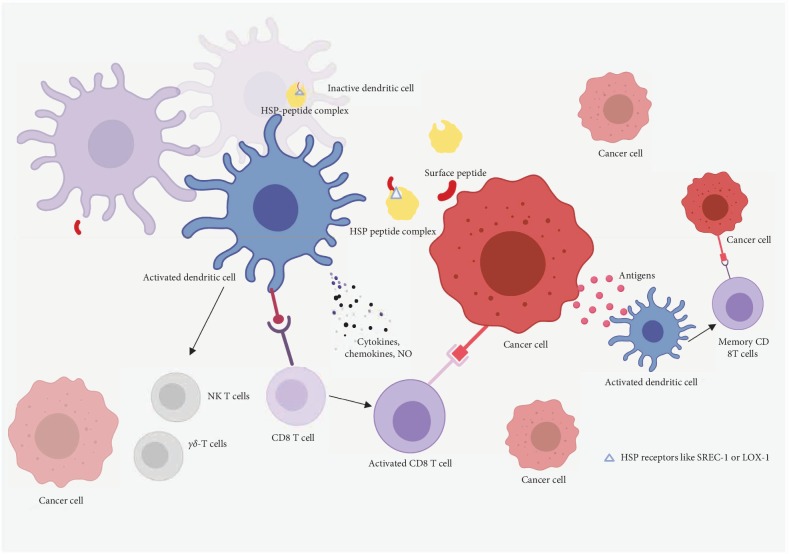
Overview of pathways in tumor antigen cross-presentation by the HSPs to the APCs. Cancer cells display limited surface peptides or antigens which are released into the extracellular milieu. These antigens are recognized by HSPs through HSP-receptors, such as SRECI and LOX-1. The HSP peptide complex may be either engulfed into the DCs through CD91 receptor-mediated endocytosis or recognized by the cognate receptors on the surface of these DCs, resulting in their activation. This leads to a cascade of subsequent innate and adaptive immunological responses against cancer cells. The activated DCs activate the *γδ* T cells and NK T cells which may facilitate the lysis of the cancer cells. These DCs also produce inflammatory cytokines, chemokines, and nitric oxide. The activation of APCs results in the recognition and killing of cancer cells through cytotoxic CD8+ T-lymphocytes response. The lysis of cancer cells releases cancer antigens into the extracellular milieu leading to the formation of memory CD8+ T cells. The cross-presentation of HSP peptide complex to APCs is therefore an effective process bridging innate and adaptive immune response and mounting an optimal anticancer immunity. The inactive DCs/CD8+ T cells are represented in light color while the activated cells are represented in dark color. This illustration has been created with Biorender.com. DC-dendritic cells.

Extracellular HSPs can bind to specific receptors on dendritic cells to promote the cross-presentation of their peptides [13]. Among the known receptors for HSPs, SRECI and LOX-1 are two of the most important receptors in this category. Stimulation of SRECI (a member of scavenger receptor family of receptors) and LOX-1 (a member of both the scavenger receptor and c-type lectin receptors family) enables the cross-presentation of HSPs with their associated peptides. LOX-1 mainly binds to HSP60 and HSP70, but SRECI binds to a wide range of common heat shock proteins including HSP60, HSP70, HSP90, HSP110, gp96, and GRP170 [14]. This cross-presentation of peptides is critical in immunosurveillance (Figure 2), as the bound peptide is not only protected against degradation but the efficiency of cross-presentation is also higher in the dendritic cells [15]. Also, the internalization of HSP-peptide complex is comparatively more efficient than the exclusive internalization of soluble antigens only. Moreover, there are very few neoantigens expressed on some tumor cells, limiting the amount of target antigens available for antigen-presentation. The cross presentation of HSP-antigenic peptide complex therefore broadens the spectrum of available HSP-peptide complexes as targets for the immune system. HSPs are also known to bind antigenic peptides displayed on cancer cells, making the HSP-peptide complexes ideal vaccination targets for cancer therapy [14]. The binding of unknown peptides by HSPs in vitro also induces peptide specific vaccine response in HSP-based anticancer immunotherapy. The success of HSP derived anticancer vaccines largely stems from their efficiency in cross-priming across the diversity of human leucocyte antigen (HLA) barrier [16]. The binding of peptides with nonpolymorphic HSPs is a process similar to the association of MHC class 1 molecules with peptides in the antigen-presentation pathway. They can make use of different HLA haplotype cases and are not limited by their specificity. This remarkable phenotype of the HSP-peptide complex makes the prospect of generating general or quasi-general HSP vaccines a distinct possibility. Some HSPs like HSP70 and HSP90 are also involved in the intracellular cytosolic pathway of cross-presentation and transportation of antigens from the endosome into the cytosol [17].

HSPs regulate the production of a range of inflammatory cytokines including TNF-*α*, IL-6, IL-10, and IL-12 [18]. The modulation of the production of these cytokines determines the anticancer as well as anti-infective immune response by the host. Besides proinflammatory and anti-inflammatory cytokines, HSPs also regulate the production of nitric oxide (NO) and chemokines [19]. Innate and adaptive immune receptors recognize and bind HSPs initiating signal transduction and production of cytokines as well as effector cells. The immunomodulatory functions of HSPs have led to their classification as “Chaperokines” or molecular chaperones. The growing evidence of the roles of HSPs in immunomodulation makes these proteins potential therapeutic targets for ameliorating immunopathies, especially metastatic immunopathies. Here, we outline the current understanding of the role of the critical HSPs, such as HSP27, HSP60, HSP70, and HSP90, in cancer therapy. We also summarize the immunomodulatory activities of HSPs and recent advances in utilization of HSPs in anticancer immunotherapy.

## 4. HSP27

HSP27 belongs to the small HSP family, and its function is regulated by phosphorylation at serine residues. The phosphorylations of Ser 15, Ser 78, and Ser 82 regulate the growth, differentiation proliferation, and migration of cells. HSP27 can reverse epithelial to mesenchymal transition and decrease the matrix metalloproteinase activity. Thus, dysregulated expression of HSP27 drives tumor development and progression. It has been shown that the expression of HSP27 is correlated with an increase in the transcription of vascular endothelial growth factor gene [20], thereby promoting angiogenesis and cellular migration in metastasis of cancer.

Several studies have shown that HSP27 is significantly higher in different malignancies such as ovarian, prostate, and breast cancer [7, 21]. HSP27 was found to be present both inside and outside the cancer cells and binds to cytochrome c, inhibiting the activation of caspases and preventing apoptosis. A study has shown that HSP27 expression was significantly correlated with the Ki-67 index in brain tumors [22]. In another study, expression of HSP27 was shown to be associated with poor prognosis in patients with meningioma [23]. The cytoplasmic immunoreactivity of HSP27 is lowered in patients with meningioma, although the chaperone protein is detected ubiquitously in all the meningioma tissues [24]. Involvement of HSP27 in tumor invasion and metastasis signaling cascades is one of the factors contributing to the poor survival rates among patients. HSP27 expression was found higher in the biopsy tissues of prostate cancer and in the serum and tumor microenvironment of female breast cancer [25]. HSP27 levels were also significantly higher in the interstitial fluid isolated from primary breast tumor tissue [26].

The structural complexity of HSPs (e.g., HSP27) makes the design of their inhibitors challenging. However, there is compelling evidence suggesting that HSP27 could be an attractive target for cancer therapy. For instance, quercetin and RP101 are two small molecule inhibitors of HSP27, which have been investigated for their anticancer properties [27, 28]. Quercetin is a bioflavonoid compound that demonstrates anticancer activity. However, currently, there are no undergoing clinical trials with quercetin, which may be due to its potent cytotoxic activity [29]. RP101 is an antiviral nucleoside that has been successfully used as an anticancer HSP27 inhibitor in clinical studies [28, 30]. Similarly, several peptide and antisense oligonucleotide inhibitors of HSP27 have been devised by several groups [31, 32], but none of them have been approved by FDA for clinical use. Therefore, the HSP27-based immunotherapeutic approaches could play an important role in the treatment of cancer. For instance, an interesting study by Straume et al. showed that HSP27 was critical in maintaining the balance between the progression and dormancy of tumor, suggesting that immunological targeting of HSP27 could be a useful strategy [8]. They showed that HSP27 was upregulated significantly in the angiogenic cells of a MDA-MB-436 breast tumor xenograft model, and those results were validated in cell lines, mouse models, and clinical datasets. They also showed that stable downregulation of HSP27 in the angiogenic tumor cells resulted in long-term tumor dormancy, and remarkably, none of the tumor cells could escape dormancy. Similarly, Mahvi et al. showed that overexpression of HSP27 in the estrogen receptor-positive MCF-7 cells stimulated the proliferation of peripheral blood lymphocytes and promoted the lysis of MCF-7 cells by *γδ* T cell clones. The role of HSP27 in modulation of vascular inflammation and chronic inflammatory disorders has been well-studied and established [33]. These studies, along with other similar evidences, indicate a great potential of the HSP27-targeted immunotherapeutic approach in treatment of cancer.

## 5. HSP60

HSP60 is an extensively studied heat shock protein, especially in the immunological context. Like other HSPs, it is an intracellular chaperone that facilitates homeostatic protein folding and transportation [34]. HSP60 is particularly well studied in the context of autoimmune diseases [35]. Self HSP60 reactive lymphocyte clones were found in healthy and physiological conditions in mammals [36, 37], demonstrating that HSP60 are indeed the key players in physiological autoimmunity. Self-HSP-reactive T and B cell clones can be categorized as significant players in immunological signal transduction pathways. These molecules control inflammation by limiting clonal expansion and are also involved in maintenance and repair of tissue. The HSP60 chaperones are thereby vital components involved in maintaining cellular homeostasis through their immunomodulatory activities [38].

HSP60 chaperone is known to play an important role in the pathogenesis of cancers. It was reported that the oncogenic HSP60 drives the development of pancreatic ductal adenocarcinoma through modulation of mitochondrial oxidative phosphorylation (OXPHOS) [39]. Tumors promoted by HSP60 were classified as “chaperonopathies by mistake,” as these molecular chaperones help promote the growth, proliferation, and metastasis of tumor cells and mediate their resistance to stressors, rather than protecting the host [40]. HSP60 is also known to be a dual regulator of apoptosis and has both pro- and antitumoral effects. Recently, a clinical study found that expressions of HSP60 and HSP70 are associated with a long-term outcome in patients with T1 high-grade urothelial bladder tumor following Bacillus Calmette–Guérin immunotherapy [41]. Also, It has been shown that immunization with a recombinant HSP60 of *Histoplasma capsulatum* elicits a protective immune response that is mediated by a subset of V*β*8.1/8.2 + T cells in a murine model [42]. Similarly, Yamazaki et al. showed that HSP60-reactive T cells accumulate in the gingival tissues of periodontitis patients [43]. Our own ongoing study has been focusing on cloning of the HSP60 reactive T-cell receptor alpha and beta chains for facilitating the directed differentiation of T lymphocytes from induced pluripotent stem cells (iPSs). Our study may have great potential to generate the HSP60-based novel immunomodulatory strategy for the treatment of various diseases including cancer.

## 6. HSP70

HSP70 is a high-molecular weight ubiquitous chaperone protein, which has a significant role in regulating cellular homeostasis, by controlling protein folding, translocation, biogenesis, and degradation [44]. Although HSP70 is primarily induced as a stress response protein, it is also constitutively expressed as a housekeeping gene in different types of cells. HSP70 contains a 44 kDa amino-terminal nucleotide binding domain with ATPase activity, 18 kD substrate binding domain, and a 10 kDa C-terminal lid [45]. HSP70 can be classified into two subfamilies: the canonical DNAk-like protein and the higher-molecular weight HSP110 [46]. The canonical HSP70s refolds misfolded proteins and suppresses protein aggregation, promoting the growth of cancer cells [47]. HSP110 members are structurally and functionally distinct from the canonical HSP70s, with a limited role in carcinogenesis, and will not be discussed in detail in this review.

The canonical HSP70 inhibits apoptosis through preventing the activation of Bax. However, HSP70 also promotes the release of proapoptotic factors and facilitates mitochondrial membrane permeabilization. Furthermore, HSP70 prevents the assembly of death inducing signaling complex (DISC) [48]. In addition, cellular senescence is induced through the p53-mediated downregulation of the canonical HSP70. In experimental models, overexpression of HSP70 was shown to increase the tumorigenicity of transformed cells, while the downregulation of HSP70 significantly decreased the tumorigenicity of the cells.

As constitutively increased expression of HSP70 leads to various types of cancers, neutralizing HSP70 has emerged as an attractive anticancer strategy. Chemotherapy increases HSP70 expression, which contributes to the resistance of cells to anticancer therapy and other cell-death inducing stimulus. Schmitt et al. have shown that a protein designated as ADD70 sensitized different human cancer cells to apoptosis by interacting with HSP70, suggesting that selective neutralization of HSP70 is beneficial in inducing apoptosis in drug-resistant cells [49]. Despite the success of targeting HSP70 as an anticancer therapeutic strategy, this approach has some limitations. For example, the inhibition of HSP70 results in undesirable cytotoxicity for normal cells, owing to its ubiquitous expression in physiological conditions. Therefore, there has been a concerted effort towards using HSP70-based targeted anticancer immunotherapy.

HSP70 anticancer vaccines have been successfully used in clinical settings with positive impact on cancer patients. For instance, a human fusion protein vaccine composed of HSP70-HPV16 oE7 antigen was shown to elicit effective CD8+ antitumor cell-mediated response [50]. Another study by Abkin et al. showed that the purified HSP70-based gel diffused effectively through the outer layer of B16 tumor, promoting intratumoral antitumor effects. Intratumorally derived HSP70 showed significant antitumor efficacy when combined with phloretin in a murine melanoma model [51]. In addition, Sato et al. reported that the leukemia cell-derived HSP70 has immunization effects and improved the survival of BALB/c mice after syngeneic bone marrow transplantation [52]. HSP70 on the cancer cell surface elevated NK cell toxicity [53] and enhanced dendritic cell maturation besides promoting the activation of T cells [54]. HSP70 is also known to initiate the functions of both the innate and adaptive immunity through the production of a range of cytokines [55]. These HSPs function as classical chaperones, enabling cross-presentation of antigenic peptides to APCs. The increased translocation of HSP70 into the extracellular milieu, which is triggered by the delivery of purified HSP70 into tumor microenvironment, enhances the sensitivity of cancer cells to conventional treatment options. The broad range of the aforementioned immunomodulatory activities of HSP70 makes it one of the most versatile HSPs for anticancer immunotherapy, with immense potential for future development.

## 7. HSP90

HSP90 is a molecular chaperone that facilitates the maturation of substrates. Several kinases, transcription factors, E3 ubiquitin ligases, and steroid hormone receptors are the partners of HSP90 and bind to HSP90 in highly dynamic conformations. The pleiotropic effects of HSP90 on several of its partner proteins implicate them in various diseases including neurodegeneration and cancer. Many of the client proteins of HSP90 are oncogenic drivers; therefore, inhibition of HSP90 is believed to have a therapeutic impact in treatment of cancer. Although the HSP90 inhibitors have been shown to be effective in solid tumors and hematological malignancies, these agents are not efficacious as stand-alone single agents in cancer patients. Combining HSP90 inhibitors with immunotherapy has been proposed as a promising strategy for exploration. For instance, Mbofung et al. have shown that inhibition of HSP90 effectively enhanced T-cell-based anticancer immunotherapy through upregulation of the interferon response genes. They observed that the combination of HSP90 inhibition with CTLA4 blockade greatly enhanced CD8+ T cell functions in tumor microenvironment [56].

Several lines of experimental evidence have established the essential role of HSP90 in antigen presentation with MHC I molecules on the cellular surface [57]. Recent studies have also shown that extracellular HSP90 binds to its peptide substrate, which is recruited by the heat shock protein receptors on antigen-presenting cells. The HSP90-peptide substrate complex is then internalized through the vesicles and processed by the proteasomes. These processed antigenic peptides are displayed on MHC II complex and released into the endoplasmic reticulum. The MHC II-peptide complex are subsequently transported to the cellular surface and presented to CD-4+ T cells, thereby activating a canonical cascade of anticancer immune response [58]. These immunological features of HSP90 indicate that inhibition of HSP90 might dampen natural immune responses, particularly anticancer immunity. Surprisingly, several independent studies have shown that HSP90 “clients proteins” like HIF-1*α* and JAK2 modulate immune-checkpoint blockade through induction of PD1 and PD-L1 expression [59, 60], suggesting that HSP90 inhibition could be used as an effective approach to enhancing anticancer immunotherapy. The efficacy of HSP90 inhibitors have since been validated in preclinical and clinical studies. Combined use of ganetespib (a HSP90 inhibitor) and STI-A1015 (an anti-PD-L1 antibody) in a syngeneic mice model bearing colon cancer or melanoma was proven to be an effective antitumor combination therapy [60]. Similarly, the HSP90 inhibitor, SNX-5422, also proved to be an effective antitumor agent when used in combination with monoclonal antibodies against PD-1, PD-L1, or CTLA4, in a colorectal cancer model system [61].

Development of anticancer vaccines has been tested with glycoprotein96 (gp96), an ER residing member of the HSP90 family of proteins. Immunogenic peptides chaperoned with gp96 were shown to elicit specific anticancer immune response, making this protein an ideal vaccine candidate. Several clinical trials in patients suffering from malignant melanoma have been conducted with gp96 chaperonic protein for testing its proposed efficacy between the years of 2000 to 2014 [62–65]. Clinical trials have also been undertaken with gp96 protein for gastric carcinoma, pancreatic carcinoma, and Hodgkin lymphoma and glioblastoma [66].

## 8. Overview of HSP70 and HSP90 Vaccines in Tumor Immunity

The HSP70 and gp96 (HSP90) vaccines are the most successful and widely used HSP vaccines.

Some common HSP70 and HSP90 vaccines are listed in Table 1. The interaction of these classical HSP vaccines with tumor immunological signaling network is complex; thus, a better understanding of this interaction is fundamental towards the development of improved and more effective anticancer HSP vaccines. Several studies have indicated that HSPs have the ability to induce T-cell tolerance, via not only shifting the cytokine response from a Th1- to a Th2-type but also promoting the suppression of the Th17 based inflammatory cytokine IL-17, with the simultaneous expansion of CD4+ CD25+ (Treg) cells [35]. The Th17 and Treg cells originate from a common precursor naïve CD4+ T cells. The disruption of the delicate Treg/Th17 balance creates an immune-suppressive environment conducive to the progression of carcinogenesis [70]. Treg cells promoted by HSPs inhibit immunological responses through the production of anti-inflammatory interleukin-10 (IL-10) cytokine. The shifting of the immune response from the inflammatory IL-17 towards the HSP driven IL 10 production may impede the mounting of an optimal immune response and induce T-cell tolerance. This environment may result in inhibition of the activity of cytotoxic T-lymphocytes and lead to the prevention of maturation of dendritic cells, thereby reducing the antigen presentation capacity [71]. However, despite these apparent limitations of using autologous and purified HSP vaccines, several HSP70 and gp96 vaccines have been successful. The interaction of HSPs with the immune system may result in a very complex outcome, which has been previously demonstrated with the inverse dose-immune response relationship of gp96 vaccines [72].

In the case of HSP70-based vaccines, the immune response is biased away from the generation of the Tregs towards the Th17-based killing of cancer cells by the CTLs [73]. Cytokines are the most potent determinants of the Treg/Th17 homeostatic balance. For instance, although the initial differentiation of Th17 and Tregs is driven by the common tumor growth factor (TGF)-*β* signal, IL-6 is critical in maintaining the subsequent homeostasis of Treg/Th17 balance [74]. Therefore, the presence of a suitable spectrum of cytokine milieu composed of IL-6 and TGF-*β* [71, 73] appears to be essential towards the functioning of HSP70 vaccines. The requirement of IL-6 for the optimal functioning of HSP70 vaccines has been demonstrated by a study showing that exclusive elevation of HSP70 was not sufficient for elimination of pancreatic tumors. In the same study, the authors found that the immune response was skewed towards the Treg cells rather than the Th17 cells [75], and this may be attributed to the lack of IL-6 in this specific tumor microenvironment. The recent developments in understanding antitumor HSP vaccine-mediated immunity has a promising potential to improve adoptive T cell transfer therapy, using patient's own T cells in conjunction with or independently of HSP anticancer vaccines, to recognize and kill tumor cells.

## 9. Conclusion

Cancer immunotherapy has gained great success as an effective therapeutic option in our fight against some malignant tumors such as melanoma. The potential of HSPs as therapeutic targets for immunotherapy has been increasingly appreciated in the past decade. Recently, several important advances have been made in the field of HSP-based oncoimmunology, including the usage of anticancer vaccines. The HSP-based anticancer vaccines have been shown to be effective against a spectrum of antigen-expressed tumors, as they not only promote the uptake of antigens by APCs but also trigger the activation of T lymphocytes. Nevertheless, improving the efficacy of penetration of the activated CTLs into tumor microenvironment remains a challenge. A better understanding of the role of HSPs in the modulation of tumor microenvironment may help greatly in designing more effective immunotherapeutic strategies. Notably, the safety and efficacy of anticancer vaccines have been improved through combination therapies, including the use of chaperone-based immunotherapy in combination with immune-checkpoint inhibitors such as the inhibitors of CTLA-4, PD-1, and PD-L1. Targeting of HSPs may also sensitize cancer cells to conventional treatments such as chemotherapy and radiotherapy. It is anticipated that the HSP-based immunotherapy shall remain a major focus in the cancer therapeutic area, with the hope for more discoveries that can be exploited as therapeutic interventions in treating patients with cancer.

## Figures and Tables

**Table 1:** Common HSP70 and HSP90 vaccines.

HSP vaccine	Target carcinoma	Clinical trial	Reference
Vitepsin-Gp96-based vaccine	Several-liver, ovarian, glioma, melanoma, etc.	Phases II and III, approved in some places.	[67]
HSP.PC 96-Gp96-based vaccine	Renal carcinoma	Phases II and III	[68]
HSP. 70PC-HSP70-based vaccine	Breast cancer	Phase I	[69]
HPV16oE7-HSP70-based vaccine	Cervical cancer		[50]
